# Increasing identification of foot at risk of complications in patients with diabetes: a quality improvement project in an urban primary health centre in India

**DOI:** 10.1136/bmjoq-2019-000893

**Published:** 2020-08-06

**Authors:** Abha Mehndiratta, Satish Chandra Mishra, Prashant Bhandarkar, Kunal Chhatbar, Francoise Cluzeau, Team PrimaryCareDoctors

**Affiliations:** 1 Global Health and Development Group, Imperial College London, London, UK; 2 Department of Surgery, WHO Collaboration Centre for Research in Surgical Care Delivery in LMIC, Bhabha Atomic Research Centre Hospital, Mumbai, India; 3 Department of Surgery, WHO Collaboration Centre for Research in Surgical Care Delivery in LMIC, BARC Hospital, Bhabha Atomic Research Centre Hospital, Mumbai, India; 4 East Deonar Dispensary, Bhabha Atomic Research Centre Hospital, Mumbai, India

**Keywords:** diabetes mellitus, electronic health records, implementation science, primary care, healthcare quality improvement

## Abstract

The majority of foot amputations are preventable in people with diabetes. Guidelines recommend that people with diabetes should receive a foot examination for risk assessment, at least annually. In an audit at a primary health centre (PHC) in Mumbai, India, no patient with diabetes was offered preventive foot assessment in preceding 12 months. Problem analysis identified a lack of clinic policy, training and equipment for foot assessment. There was no standardised referral pathway for patients identified with foot at risk of diabetes complications. Furthermore, limited data review, high patient volumes and little time available with healthcare providers were important constraints. A quality improvement project was carried out at the PHC from January to September 2017. The project aimed at increasing compliance to standardised foot assessment in patients with diabetes presenting to the PHC from a baseline of 0% to 100% over 6 months. This would help identify patients having a foot at risk of complications due to diabetes. The Quality Standard on foot assessment was adopted from the Ministry of Health and Family Welfare Diabetic Foot Guideline. The electronic medical record (EMR) was standardised, health providers were trained, PHC processes and referral pathways were redesigned. Plan-Do-Study-Act was used to address barriers with weekly data review. 88.2% (848) of patients with diabetes visiting the PHC during the study period received a foot examination. Out of these, 11% (95) were identified to have a foot at risk and referred to a specialist foot centre. 57% of referred patients followed with specialised foot protection services. Training of healthcare providers, standardisation of processes and regular data feedback can improve diabetic foot care. Integrating quality indicators in the EMR helps monitor compliance. The inability to use doctor’s time efficiently was the biggest challenge and sustaining the change will require organisational changes with suitable task shifting.

## Problem

Studies from India suggest that foot examination in people with diabetes is not carried out routinely unless the patient reports a foot problem.^1^ East Deonar Dispensary is one of the 13 primary health centres (PHC) attached to the Bhabha Atomic Research Centre (BARC) Hospital in Mumbai. BARC Hospital is a 390-bedded multispecialty referral hospital that provides services under the Contributory Health Service Scheme of Department of Atomic Energy, Government of India. The Department of Surgery at BARC Hospital with 55 beds provides both foot protection services and tertiary care management of foot complications. The East Deonar Dispensary is managed in two shifts of 6 hours each by 2–3 primary care doctors in each shift and it has an average daily outpatient load of 270 patients (average 6500 patients per month). In addition, each shift has two nurses, two pharmacists, two general duty workers and one data entry operator. On 1 April 2017, the Dispensary had 12 017 registered patients and 1015 (8.4%) of these were on medication for diabetes mellitus. The baseline review of records showed that preventive foot examination had not been offered to any patient with diabetes in the previous 12 months. In the previous year (2016), the Department of Surgery received a total of 86 referrals from all the 13 PHCs for the management of foot ulcers in patients with diabetes. There was no separate community podiatry or a system for identification and referral of patients to foot protection services at the Department of Surgery.

The project aimed to increase compliance to standardised foot assessment in patients with diabetes presenting to East Deonar Dispensary from a baseline of 0% to 100% from 1 April to 30 September 2017. This would help stratify all patients with diabetes, receiving a foot assessment, into risk categories according to the International Working Group on Diabetic Foot (IWGDF) guideline.^2^

## Background

India with its steeply rising prevalence of diabetes is set to become the diabetes capital of the world with reported 69.2 million people with diabetes in 2015 (prevalence rate 8.8%) and a projected 123.5 million by 2040.^3 4^ Foot complications are one of the most serious complications of diabetes which cause immense psychological, social and economic consequences and may lead to an amputation.^5^ The leading cause of lower-limb amputations is diabetes and 40%–85% of these are preventable.^6 7^ According to 2010 estimates, around 45 000 legs are amputated every year in India due to diabetes, of which 75% are potentially preventable as they result from an infected neuropathic foot.^8^ The key to the prevention of foot complications and amputations in patients with diabetes is early recognition to identify ‘at-risk’ groups so that they can be offered patient education, preventive podiatry, appropriate footwear, early surgical intervention if necessary and continuous follow-up.^9^

Indian and international guidelines recommend that people with diabetes should have their feet checked at least once a year.^10 11^ Primary care providers, with their sustained relationship with patients, are best placed to identify foot at risk of complications due to diabetes.^12–15^ Surveys however indicate that compliance to standard recommendations of annual foot examination in patients with diabetes in primary care is dismal.^16–19^

Reported barriers to preventive services in primary care include lack of training and resources, lack of time, burden of acute illnesses, clinical inertia, unrewarding administrative tasks, lack of incentives for preventive care, lack of accountability and poor organisational structure.^20–24^

Implementing and sustaining change in clinical practice is notoriously challenging even if clinicians agree with the evidence, and non-adherence to evidence-based guidelines is widely reported in preventive care management of chronic diseases.^1 25 26^ A variety of strategies have been used to improve the quality of preventive care delivery, which include financial incentives (pay for performance), training of healthcare providers, audit and feedback, task shifting to non-physician healthcare providers, use of measurable quality indicators, organisational changes, etc. Among these, the organisation of care and audit feedback are reported to have a sustainable effect.^27^ Such preventive strategies are usually successful with organisational support and a computerised tracking system is used for audit feedback.^28^

This article reports on improving preventive foot care practice for patients with diabetes through implementing local Quality Standard (QS) in a busy PHC in urban India by providing training, onsite mentoring, redesigning of care pathways, standardisation of electronic medical records (EMR), regular internal audit and feedback and addressing barriers using Plan-Do-Study-Act (PDSA) for rapid-cycle testing.^29^

## Measurement

A baseline assessment was conducted by a multidisciplinary quality improvement (QI) team consisting of three primary care physicians, one nurse, two surgeons, one QI expert, a registration clerk and a statistician. EMR of all patients registered with East Deonar Dispensary for 12 months before the start of the project implementation were reviewed and patients with diabetes were identified. The American Diabetes Association defines the diagnostic criteria for type 2 diabetes as fasting plasma glucose (FPG) 126 mg/dL or glycosylated haemoglobin (A1C) ≥6.5%, confirmed by a repeat testing.^30^ However, for this study the operational definition was an adult with age 18 years and above and receiving at least one medicine or insulin for the management of diabetes (except gestational diabetes). This operational definition was chosen for the following reasons. (1) Among the dispensary doctors, there was a variation in cut-off points of A1C in diagnosing diabetes. (2) There was variation in data entry in EMR and diagnosis was missing in many records making it impossible to identify the patients with diabetes directly from the Hospital Information System (HIS). (3) Laboratory values of A1C or FPG could not be used to diagnose diabetes as EMR was introduced in 2008 and it was not possible to retrieve laboratory records before that period.

Pharmacy dispensing records from HIS were used to identify and track patients on at least one medicine or insulin for the management of diabetes. A manual review of the list was carried out by dispensary doctors to exclude patients receiving an antidiabetic drug for any other diagnosis, for example, metformin for polycystic ovarian disease. Our operational definition may have missed out some patients with early diabetes who were not on treatment. However, foot complications are known to occur in people with long-standing diabetes and it is unlikely that the patients we may have missed had a foot at risk of complications.^31^

After patients with diabetes were identified, their EMR was reviewed to find out if they had received a foot examination for risk assessment in 12 months preceding the QS implementation. The baseline review of records showed that none of these patient had received a foot examination. In preparation for the QI project, the recording of foot assessment in patients with diabetes was standardised in the EMR by introducing a prefilled format with a dropdown menu and a process was created for patients with diabetes to have their diagnosis recorded along with their IWGDF risk category on the cover page of their EMR. Furthermore, a process was created to track the follow-up of referred patients to the specialist foot protection clinic using the EMR. Finally, a process was established to regularly collect data on compliance with the standardised protocol for foot assessment. This included a manual daily data collection by the registration clerk and a mechanism for a statistician to retrieve data from the HIS every week. This data collection entailed a listing of patients with diabetes visiting the dispensary based on two sources: (1) the registration clerk identifying patients with diabetes by enquiring with them at the time of registration and (2) a daily review of the pharmacy records to identify patients who were issued drugs to control diabetes. Records of patients on this list were then manually reviewed to check whether a foot assessment had been performed and recorded. From the HIS, similar information was retrieved every week. The dual system of data collection was kept in place initially to have a redundancy while the data collection process was being streamlined. The following process and outcome measures were used to assess and monitor improvement.

Process measure:

(i) The proportion of patients with diabetes attending the dispensary who had not received a standardised foot risk assessment in the preceding 12 months and received it during that visit.

Outcome measure:

(i) The proportion of patients with diabetes identified to have a foot at risk for complications following standardised foot examination.

## Design

**Figure 1:**
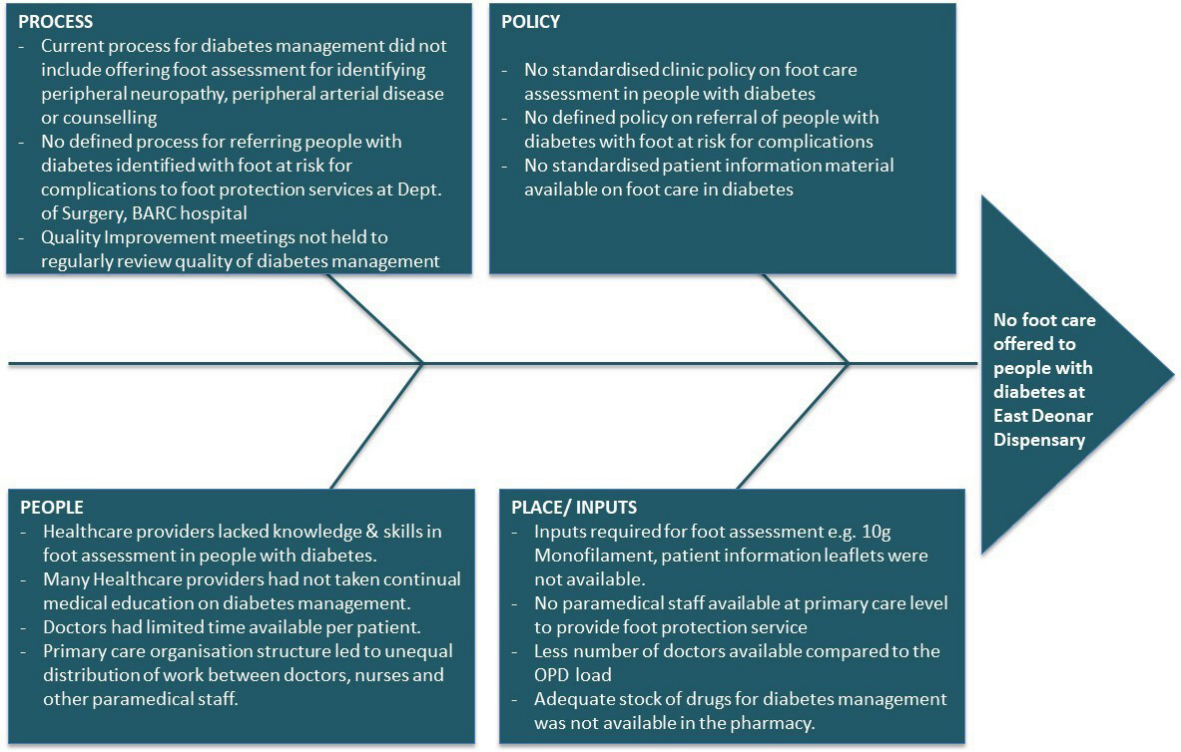
Problem analysis by Ishikawa diagram. OPD, Out Patient Department.

The QI team assessed the problem using Fishbone analysis (Ishikawa diagram) (figure 1).^32^ The causes identified included: lack of a clinic policy to offer annual foot examination to patients with diabetes; primary care doctors not trained in diabetic foot (DF) care; lack of equipment for foot examination; patient data not reviewed and used for regular feedback; absence of a standardised referral pathway for patients identified with a foot at risk and limited time available with a doctor for each patient due to high patient volume.

**Figure 2:**
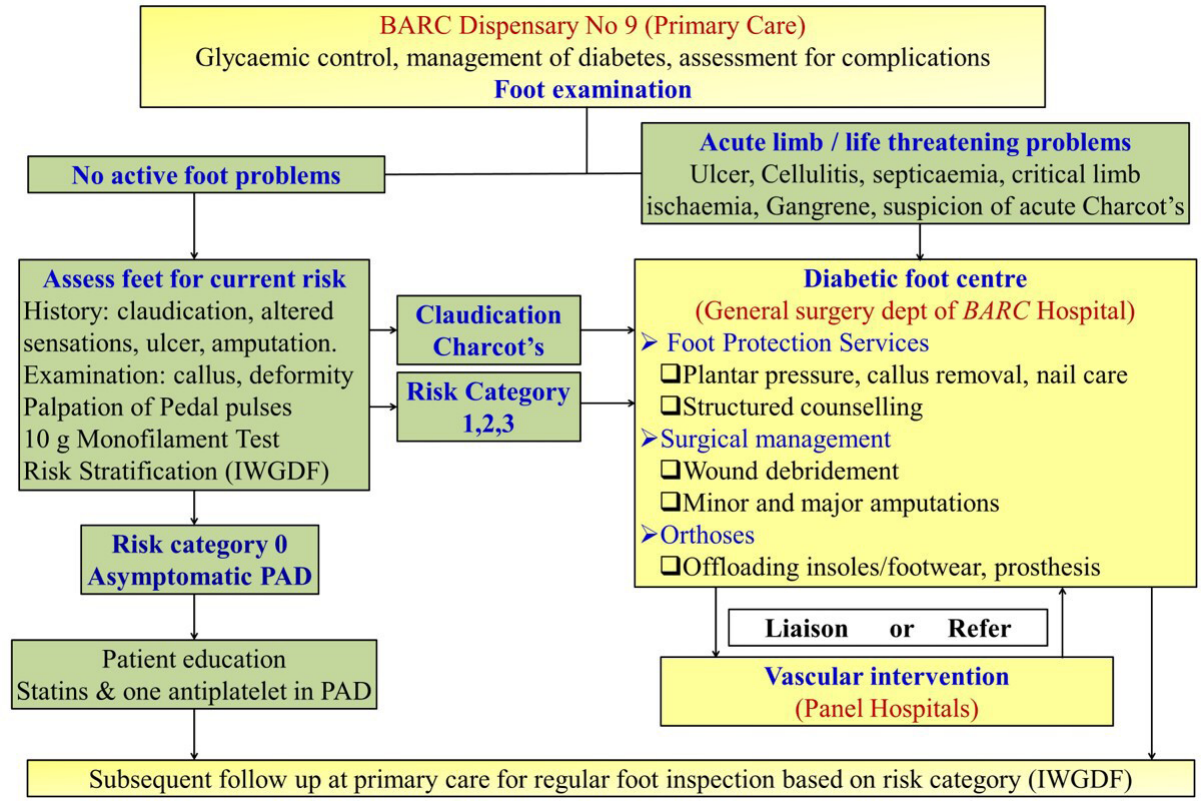
Redesigned care pathway for diabetic foot care. PAD, peripheral arterial disease; IWGDF, International Working Group on Diabetic Foot.

The QS from the Ministry of Health and Family Welfare guideline on ‘The Diabetic Foot’ was adapted into a standardised clinic policy for foot assessment in patients with diabetes. It was contextualised for East Deonar Dispensary and BARC healthcare system.^10^ The policy included details on when and how to assess feet of patients with diabetes for risk factors, stratification of risk, counselling on foot care, appropriate referral and further follow-up (figure 2). According to this policy, all patients with diabetes visiting the dispensary who had not received a standardised foot risk assessment in the last 12 months should receive a foot evaluation. The protocol included eliciting a history of altered sensation, claudication, past ulcers or amputations and a clinical examination for callus, deformity, webspace infection, peripheral arterial disease (PAD) and peripheral neuropathy (PN). PAD was assessed by clinical history and palpation of posterior tibial artery (PTA) and dorsalis pedis artery (DPA) of the foot. PN was assessed by loss of protective sensation (LOPS) using a Semmes-Weinstein’s 5.07 monofilament (standardised screening tool). Based on this evaluation, patients were stratified into risk categories (0–3) according to the IWGDF guideline (table 1).^2^ Patients found to have claudication or risk categories 1–3 were referred to foot protection service at the Department of Surgery of BARC Hospital. The foot protection service provided plantar pressure assessment, callus removal and appropriate footwear to offload pressure points in consultation with a pedorthotist. Patients with asymptomatic PAD were offered statins and one antiplatelet. All patients were further followed at the PHC as per the IWGDF guideline described in table 1.^2^

**Table 1:** Risk classification system and preventive assessment frequency^2 10^

Category	Characteristic	Follow-up frequency
0	No peripheral neuropathy	Once in a year
1	Peripheral neuropathy	Once in every 6 months
2	Peripheral neuropathy with peripheral artery disease and/or a foot deformity	Once in every 3–6 months
3	Peripheral neuropathy and history of a foot ulcer or lower-extremity amputation	Once in every 1–3 months

Training was provided to primary care doctors and nurses on the standardised policy for foot assessment in patients with diabetes. Also a refresher training on the management of diabetes in primary care was included since variation in clinical practice was noted. The supplies needed for foot assessment and patient education in primary care were assessed and procured.

A written informed consent was obtained from each patient before they were included in the project.

## Strategy

A series of PDSA cycles were tested over 6 months to improve foot examination among patients with diabetes. The QI team met regularly to review the analysed data, discussed barriers as they arose and designed solutions to address the problems identified. Data were shared and discussed with front-line staff and patient representatives. The key PDSA cycles tested during this period are listed in the attached online supplementary file 1.

10.1136/bmjoq-2019-000893.supp1 Supplementary data

PDSA 1: A new clinic policy on standardised foot assessment of patients with diabetes was developed by the QI team. It was adapted from the MoHFW guideline and contextualised for the BARC healthcare system. This policy was introduced from 1 April 2017 and data collected on how many doctors were complying with it. Only three out of six primary care doctors were found to comply with the new policy because some of the doctors still lacked confidence in their skills to assess the feet of patients with diabetes. The initial training primary care doctors had received was perceived to be theoretical and lacked emphasis on practical skills. Hence, onsite skill-building training was suggested.

PDSA 2: Onsite Training was provided by the foot protection team of BARC Department of Surgery and data were collected to validate foot assessment findings of primary care doctors by a repeat examination done by a general surgeon. Variation in assigned risk categories was noted between primary care doctors and the surgeon due to different instructions provided to the patient before the examination. This led to a discrepancy in patient response at the time of foot assessment. Also, patients were examined in variable positions ranging from sitting, reclining to lying down. In addition, there was variation in the technique used to perform monofilament test and palpating peripheral pedal pulses. Continued onsite mentorship by a general surgeon was proposed to standardise foot assessment, decrease interobserver variation and strengthen the skills of primary care doctors.

PDSA 3: Regular onsite mentorship continued to standardise patient instructions, position during the examination, the technique of monofilament test and palpation of peripheral pulses. This was provided by a general surgeon for 1 hour per week for six consecutive weeks. All doctors agreed to change the examination position to lying down for a more reliable foot assessment. Also, the number of points to be assessed for monofilament test was standardised to 10 points (nine plantar and one dorsal) and the definition of LOPS was standardised as ‘lack of sensation at even a single point, confirmed by retesting the site’.^33^ Furthermore, PAD was defined as the absence of pulsation in both the DPA and PTA. Data were again collected to validate foot assessment findings and a decrease in variation was noted in assigned risk categories between primary care doctors and surgeons. Primary care doctors with better skills were assigned management of all patients with diabetes during their shift. They also took on the responsibility of providing mentorship to their peers.

PDSA 4: The QI team assigned one physician in every Out Patient Department (OPD) shift to provide management to all patients with diabetes, including foot assessment and counselling. Data collected showed an increase in the proportion of patients who had received a foot examination. However, an uneven distribution of work was noted. There are usually 2–3 doctors in every OPD shift. Since one doctor was providing care only to the patients with diabetes, which accounted for <10% of all patients registered with the dispensary, the remaining 1–2 doctors had a huge patient load. Hence, daily foot assessment was stopped and replaced by a once a week dedicated diabetes clinic.

PDSA 5: Saturday was chosen for the dedicated diabetes foot clinic. This is because on Saturday the Dispensary had an existing process of offering glucometer blood sugar testing to monitor diabetes control and a single shift with six doctors. Two out of six doctors were assigned to provide diabetes management including foot assessment. However, this change caused a decrease in the proportion of patients with diabetes who received foot examination in that week because many patients visited the clinic on Monday–Friday only and therefore missed a foot examination. To address the issue, daily diabetes management was also offered on weekdays for patients unable to attend the dedicated diabetes clinic on Saturdays. To increase the proportion of patients with diabetes receiving foot examination, the QI team suggested testing a whole day diabetes camp.

PDSA 6: Whole-day special diabetes camp was organised by the dispensary in collaboration with the Surgery Department foot protection team. Data continued to be collected on the proportion of patients with diabetes who received a foot examination. Patients appreciated receiving multidisciplinary services in one place, and the proportion of patients with diabetes receiving foot examination increased. The QI team agreed to continue offering a dedicated whole-day diabetes camp once in every 1–2 months. This was provided along with the weekly (Saturday) diabetes clinic and daily diabetes management.

While testing PDSAs, a random audit of the manual data collection process revealed errors. The reasons for errors were as follows. (1) Some patients with diabetes could not be identified by the registration clerk during rush hours or if patients were unwilling to share their diabetes status. (2) Some patients with diabetes came to the dispensary for a treatment other than a refill of diabetic medication and therefore could not be identified by a review of pharmacy records. (3) There were few duplicate entries due to human errors, and unique patient identifiers helped detect them. As a result, a greater reliance was shifted to data retrieved from HIS and manual data collection was subsequently discontinued.

## Results

At the start of the study, on 1 April 2017 the East Deonar Primary Health Centre had a total of 12 017 registered patients. By the end of the study period on 30 September 2017, 1515 patients had moved out of the primary care centre (emigrant population) due to work transfer or retirement, leaving 10 502 patients on the list. Meanwhile, 1629 new patients were registered due to transfer or recruits (immigrant population). This gave a total of 13 646 baseline population for the study. From this, 1087 (7.9%) patients were identified to have diabetes.

Of note, 961 (88.4%) patients with diabetes visited the PHC at least once in the 6 months during the QS implementation. Among these, 848 (88.2%) received a foot assessment. Out of 848 patients who had their feet examined, 95 (11%) were identified to have a foot at risk (RC1–85, RC2–5, RC3–5). LOPS was present in all 95 (11%) patients and PAD was found in 10 (1.2%) patients. All patients with a foot at risk of complications were referred to a specialist foot clinic. However, only 54/95 (57%) patients followed up. The results are summarised in table 2.

**Table 2:** Result of foot examination in patients with diabetes visiting East Deonar Dispensary

Clinic population	Total no. of patients with diabetes	No. (%) of patients with diabetes who visited East Deonar Dispensary at least once during the study period	No. (%) of patients with diabetes who had their feet examined once during the study period	No. (%) of patients assigned risk categories after foot examination				No. (%) of patients referred to specialty diabetes foot clinic	No. of patients (%) followed at specialty diabetes foot clinic
				RC0	RC1	RC2	RC3		
Total, 13 646	1087 (7.9%)	961 (88.4%)	848 (88.2%)	753 (89%)	85 (10%)	5 (0.6%)	5 (0.6%)	95 (100%)	54 (57%)
Baseline population on 31 March 2017–2010, 502	888 (8.5%)	826 (93.%)	736 (89.1%)	650 (88%)	77 (10.5%)	5 (0.6%)	4 (0.3%)	86 (100%)	50 (58%)
Immigrant population 31 March–30 September 2017, 1629	72 (4.4%)	14 (19.4%)	14 (100%)	14 (100%)	0	0	0	0	0
Emigrant population after 31 March–30 September 2017, 1515	127 (8.4%)	98 (77.2%)	98 (100%)	89 (91%)	8 (8%)	0	1 (1%)	9 (100%)	9 (100%)

## Lessons and limitations

The key lessons learnt and limitations encountered during this QI project are as follows:

1. PN, defined by LOPS to 10 g monofilament, was found in 11% of patients with diabetes which is consistent with the reported range of 8.4%–17.7% in similar clinical settings (patients with diabetes reporting at community centre) using the same detection tool (10 g monofilament).^34 35^ The QI project achieved a foot assessment in 88.2% of patients with diabetes. Many of the patients who did not have their feet examined were actually ‘proxy consultations’ with family members sent to collect medicines on their behalf. We need a plan to reach out to patients who are elderly or live far away and send a representative to collect medicines on their behalf. Despite repeated reminders, we failed to get these patients to visit the clinic for foot evaluation.
2. Contextually appropriate refresher training of primary care physicians needs to be combined with ongoing coaching and mentoring to build knowledge, skills and confidence.
3. A team-based approach with continuous engagement of healthcare providers was useful in reviewing data, identifying problems and planning changes tested through the ‘PDSA’ cycles to standardise the process of foot assessment in patients with diabetes. There were frequent team meetings to review data, celebrate success and address barriers. Intrinsic motivation was built in primary care physicians when they received appreciative feedback from patients and peers. This was key to the success of the project.
4. The use of simple measures to track progress and integration of quality indicators in existing medical records helped in the improvement of processes.^1 36^ Improvements were noted using a system of regular audit, feedback and re-organisation of care pathways.^37 38^
5. Support by dispensary leadership was critical to remove barriers, test new changes, institutionalise new processes and keep the dispensary staff motivated. Strong and competent clinical leadership is critical to give the initial boost to any change in a system riddled by clinical inertia, address local barriers and ensure teamwork.^38^
6. The doctor’s time was noted to be inefficiently used and was a key bottleneck. A large proportion of the doctor’s time was spent on non-clinical clerical tasks such as organising patient appointments for specialists. They also provided frequent medicine refills because pharmacy had limited stocks and were required to dispense prescriptions for short duration only. This barrier requires system changes that are beyond the authority of the PHC leadership. In the absence of these organisational changes, the primary care doctors are at risk of a ‘burn-out’ with little interest in preventive care. It is acknowledged that improvement in preventive care has more to do with the organisation of care than gaps in knowledge or skills.^38^
7. Another challenge was the difficulty in providing one-to-one structured patient education on diabetes during a busy clinic. Doctors found detailed counselling very time-consuming and a hurried one-sided patient education proved unrewarding. Task-shifting responsibilities from doctors to nurses was attempted for counselling of patients. However, nurses did not report to the local dispensary leadership and wider system-level changes were required to change their defined roles and responsibilities. As an alternative, patient education handouts were distributed while patients waited for their doctor’s consultation. These handouts were prepared in English and in two other regional languages. They emphasised self-care, identification of warning signs and whom to report. Also, doctors running the Saturday Diabetes Clinic started group education and interactive sessions with the help of audiovisual aids including patient education videos. The one-to-one structured patient education was limited to patients with risk categories 1–3 who followed up for foot protection services at the Department of Surgery.
8. It is well known that preventive activities are often not taken seriously by patients especially if they are asymptomatic.^39 40^ Out of the 95 patients with a foot at risk referred from the PHC to the foot protection service, only 57% followed up despite telephonic reminders.
9. An unintended positive consequence was that overall management of diabetes improved along with an improvement in foot care. This is because providers received refresher training on the management of diabetes in primary care and started regularly reviewing their data, and there was an increased focus on diabetes management with the weekly Saturday clinic and monthly camp.
10. No systematic evaluation of costs was conducted in the implementation of this new primary care practice of assessing the feet of patients with diabetes. Further studies on both cost and cost-effectiveness would be helpful.

While the QI project was successful in meeting its initial objective of assessing a substantial proportion of patients with diabetes for identification of foot at risk of complications, sustaining the clinical practice as a routine is still a challenge. The QI team would need to continue regular and repetitive audit and feedback, combined with other system-level interventions to address barriers.^37^

## Conclusion

To the best of our knowledge, this is the first time QS derived from Standard Treatment Guideline on Diabetic Foot has been implemented in primary care in India. In this pilot project, we were able to create an integrated multidisciplinary DF care service but this may be difficult to replicate in other primary care settings. There is currently limited capacity in India and the South Asia region to provide specialised DF care services. In low/middle-income countries (LMICs), podiatry as a discipline is emerging, and few tertiary care centres are providing multidisciplinary foot care services. DF complications are usually treated in the general surgery units of secondary or tertiary care hospitals.^33^ LMICs need to progress and develop a three-tier system for foot care similar to advanced healthcare systems in high-income countries. This includes preventive services and appropriate referral from primary care; foot protection services at community level for management of simple foot problems and multidisciplinary foot care services at tertiary level to handle complex foot problems.^11^ In our project, reliable use of data for continuous QI was possible because of the availability of EMR. This may however not be immediately generalisable to health systems in India which are still dependent on paper records. A national commitment to introduce EMR in the public health system has been made but it is likely to take time to be implemented. Integrating quality indicators in the EMR would help monitor the quality of care delivered.

## References

[1] Harrison-Blount M, Cullen M, Nester CJ, et al The assessment and management of diabetes related lower limb problems in India-an action research approach to integrating best practice. J Foot Ankle Res 2014;7:1–9. 10.1186/1757-1146-7-30 24862010 PMC4032386

[2] Bakker K, Apelqvist J, Lipsky BA, et al The 2015 IWGDF guidance documents on prevention and management of foot problems in diabetes: development of an evidence-based global consensus. Diabetes Metab Res Rev 2016;32:2–6. 10.1002/dmrr.2694 26409930

[3] IDF diabetes atlas - 2015 Atlas International diabetes federation, Brussels, Belgium 2015.

[4] Pradeepa R, Mohan V Prevalence of type 2 diabetes and its complications in India and economic costs to the nation. Eur J Clin Nutr 2017;71:816–24. 10.1038/ejcn.2017.40 28422124

[5] Jeffcoate W, Bakker K World diabetes day: footing the bill. Lancet 2005;365:1527. 10.1016/S0140-6736(05)66437-9 15866295

[6] Lavery LA, Armstrong DG, Vela SA, et al Practical criteria for screening patients at high risk for diabetic foot ulceration. Arch Intern Med 1998;158:157. 10.1001/archinte.158.2.157 9448554

[7] Centers for Disease Control and Prevention National diabetes statistics report: estimates of diabetes and its burden in the United States. US Dep Heal Hum Serv 2014:2014.

[8] Pendsey S Clinical profile of diabetic foot in India. Int J Low Extrem Wounds 2010;9:180–4. 10.1177/1534734610380025 21134956

[9] U.S. Department of Health and Human Services Healthy people 2000: National health promotion, disease prevention objectives. 24 Washington, DC: US Department of Health and Human Services, Public Health Service, 1990; [DHHS publication no. (PHS)91-50212.], 1990: 459.

[10] Team SD foot Standard treatment guidelines: The Diabetic Foot - Prevention and management in India 2016. Website Minist Heal Fam welfare, India, 2016 Available: http://clinicalestablishments.nic.in/En/1068-standard-treatment-guidelines.aspx

[11] Team ICG NICE clinical guideline no 19. diabetic foot problems?: prevention and management. Diabet Foot Probl Prev Manag 2016.

[12] Grumbach K, Bodenheimer T A primary care home for Americans. JAMA 2002;288:889–93. 10.1001/jama.288.7.889 12186609

[13] Donaldson MS, Karl D, et al Vanselow. Editors; Committee on the future of primary care I of medicine. Primary care?: America's health in a new era. National Academy press. Washington DC. Washington, D.C: National Academies Press, 1996: 3–6. 25121221

[14] Del Aguila MA, Reiber GE, Koepsell TD How does provider and patient awareness of high-risk status for lower-extremity amputation influence foot-care practice? Diabetes Care 1994;17:1050–4. 10.2337/diacare.17.9.1050 7988305

[15] Safran DG Defining the future of primary care: what can we learn from patients? Ann Intern Med 2003;138:248–55. 10.7326/0003-4819-138-3-200302040-00033 12558375

[16] Wylie-Rosett J, Walker EA, Shamoon H, et al Assessment of documented foot examinations for patients with diabetes in inner-city primary care clinics. Arch Fam Med 1995;4:46–50. 10.1001/archfami.4.1.46 7812476

[17] Alonso-Fernández M, Mediavilla-Bravo JJ, López-Simarro F, et al Evaluation of diabetic foot screening in primary care. Endocrinol Nutr 2014;61:311–7. 10.1016/j.endoen.2014.06.008 24582291

[18] Levitt NS, Bradshaw D, Zwarenstein MF, et al Audit of public sector primary diabetes care in Cape Town, South Africa: high prevalence of complications, uncontrolled hyperglycaemia, and hypertension. Diabet Med 1997;14:1073–7. 10.1002/(SICI)1096-9136(199712)14:12&lt;1073::AID-DIA498&gt;3.0.CO;2-9 9455936

[19] Bovier PA, Sebo P, Abetel G, et al Adherence to recommended standards of diabetes care by Swiss primary care physicians. Swiss Med Wkly 2007;137:173–81. doi:2007/11/smw-11592 17457700 10.4414/smw.2007.11592

[20] Sommers LS et al A descriptive study of managed-care hassles in 26 practices. West J Med 2001;174:175–9. 10.1136/ewjm.174.3.175 11238348 PMC1071306

[21] Yarnall KSH, Pollak KI, Østbye T, et al Primary care: is there enough time for prevention? Am J Public Health 2003;93:635–41. 10.2105/AJPH.93.4.635 12660210 PMC1447803

[22] Phillips LS, Branch WT, Cook CB, et al Clinical inertia. Ann Intern Med 2001;135:825–34. 10.7326/0003-4819-135-9-200111060-00012 11694107

[23] Nam S, Chesla C, Stotts NA, et al Barriers to diabetes management: patient and provider factors. Diabetes Res Clin Pract 2011;93:1–9. 10.1016/j.diabres.2011.02.002 21382643

[24] Funk SG, Champagne MT, Wiese RA, et al Barriers to using research findings in practice: the clinician's perspective. Appl Nurs Res 1991;4:90–5. 10.1016/S0897-1897(05)80062-X 1741642

[25] Lesho EP, Myers CP, Ott M, et al Do clinical practice guidelines improve processes or outcomes in primary care? Mil Med 2005;170:243–6. 10.7205/MILMED.170.3.243 15828703

[26] Worrall G, Chaulk P, Freake D The effects of clinical practice guidelines on patient outcomes in primary care: a systematic review. CMAJ 1997;156:1705–12. 9220922 PMC1227585

[27] Jamtvedt G, Young JM, Kristoffersen DT, et al Audit and feedback: effects on professional practice and health care outcomes. Cochrane Database Syst Rev 2006;2:CD000259. 10.1002/14651858.CD000259.pub2 16625533

[28] Kirkman MS, Williams SR, Caffrey HH, et al Impact of a program to improve adherence to diabetes guidelines by primary care physicians. Diabetes Care 2002;25:1946–51. 10.2337/diacare.25.11.1946 12401737

[29] Taylor MJ, McNicholas C, Nicolay C, et al Systematic review of the application of the plan-do-study-act method to improve quality in healthcare. BMJ Qual Saf 2014;23:290–8. 10.1136/bmjqs-2013-001862 PMC3963536 24025320

[30] American Diabetes Association Diagnosis and classification of diabetes mellitus. Diabetes Care 2010;33:S62–9. 10.2337/dc10-S062 20042775 PMC2797383

[31] Al-Rubeaan K, Al Derwish M, Ouizi S, et al Diabetic foot complications and their risk factors from a large retrospective cohort study. PLoS One 2015;10:e0124446. 10.1371/journal.pone.0124446 25946144 PMC4422657

[32] Ishikawa K Introduction to quality control. Chapman-Hall, 1989.

[33] Mishra SC, Chhatbar KC, Kashikar A, et al Diabetic foot. BMJ 2017;359:j5064–7. 10.1136/bmj.j5064 29146579 PMC5688746

[34] Rith-Najarian SJ, Stolusky T, Gohdes DM Identifying diabetic patients at high risk for lower-extremity amputation in a primary health care setting. A prospective evaluation of simple screening criteria. Diabetes Care 1992;15:1386–9. 10.2337/diacare.15.10.1386 1425105

[35] Scottish Diabetes Survey Monitoring Group Scottish diabetes survey 2014. NHS Scotland, 2014: 1–82.

[36] Al-Ubaidi BA, Al-Khadraji MA, Al-Hermi B Measuring adherence rate to quality indicators for diabetes care identified by primary health care in Bahrain. Saudi Med J 2014;35:975–80. 25228179

[37] Ivers N, Jamtvedt G, Flottorp S, et al Audit and feedback: effects on professional practice and healthcare outcomes. Cochrane Database Syst Rev 2012;6:1–227. 10.1002/14651858.CD000259.pub3 PMC11338587 22696318

[38] Mash R, Levitt NS, Van Vuuren U, et al Improving the annual review of diabetic patients in primary care: an appreciative inquiry in the Cape Town district health services. South African Family Practice 2008;50:50–50d. 10.1080/20786204.2008.10873764

[39] Ornstein SM, Musham C, Reid A, et al Barriers to adherence to preventive services reminder letters: the patient's perspective. J Fam Pract 1993;36:195–200. 8426139

[40] Rotarou ES, Sakellariou D Determinants of utilisation rates of preventive health services: evidence from Chile. BMC Public Health 2018;18:839. 10.1186/s12889-018-5763-4 29976166 PMC6034328

