# Supplementary figures and images for: Increasing identification of foot at risk of complications in patients with diabetes: a quality improvement project in an urban primary health centre in India
Source: BMJ Open Qual. 2020 Aug 6;9(3):e000893. doi: 10.1136/bmjoq-2019-000893 (PMC7412605; doi:10.1136/bmjoq-2019-000893)

## PDSA CYCLES

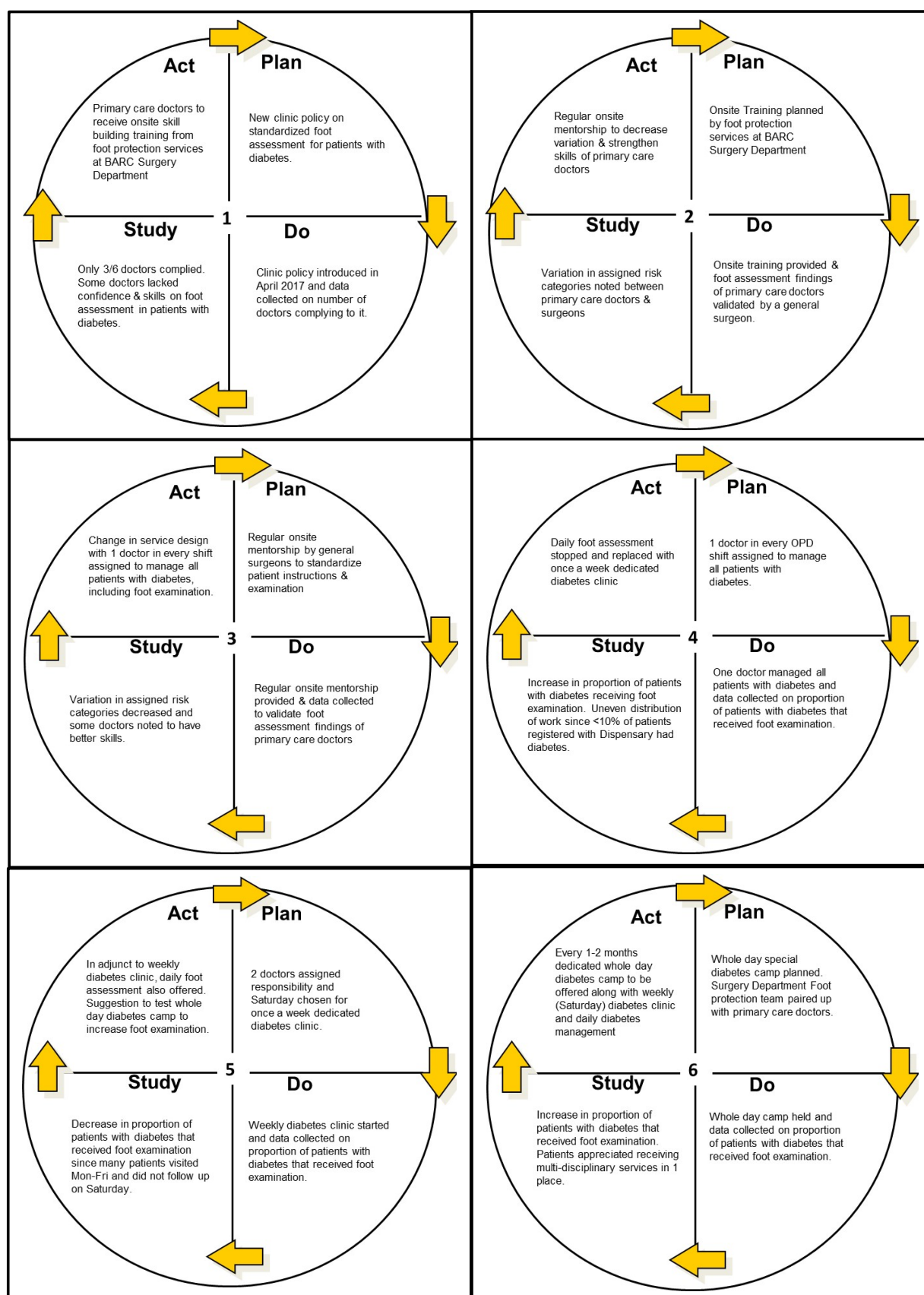

Supplement: Supplementary data [file bmjoq-2019-000893supp001.pdf]
